# The Current State of Breast Cancer Genetics in Populations of African Ancestry

**DOI:** 10.3390/genes16020199

**Published:** 2025-02-06

**Authors:** Sarah Elisabeth Santos Cupertino, Ana Carolina Aparecida Gonçalves, Claudemira Vieira Gusmão Lopes, Daniela Fiori Gradia, Marcia Holsbach Beltrame

**Affiliations:** 1 Programa de Pós-Graduação em Genética, Departamento de Genética, Universidade Federal do Paraná (UFPR), Centro Politécnico, Jardim das Américas, Curitiba 81531-980, Paraná, Brazil; sarah.elisabeth@ufpr.br (S.E.S.C.); danielagradia@ufpr.br (D.F.G.); 2 Laboratório de Genética Molecular Humana, Departamento de Genética, Universidade Federal do Paraná (UFPR), Centro Politécnico, Jardim das Américas, Curitiba 81531-980, Paraná, Brazil; ana.goncalves1@ufpr.br; 3 Câmara de Educação do Campo, Universidade Federal do Paraná (UFPR), Setor Litoral, Matinhos 83260-000, Paraná, Brazil; claudemira@ufpr.br; 4 Laboratório de Citogenética Humana e Oncogenética, Departamento de Genética, Universidade Federal do Paraná (UFPR), Centro Politécnico, Jardim das Américas, Curitiba 81531-980, Paraná, Brazil

**Keywords:** breast cancer subtypes, African diaspora, health disparities, admixed populations, Brazilian population

## Abstract

Breast cancer (BC) constitutes a significant global health burden, particularly among women, with disparities observed across populations. Notably, women of African ancestry often experience BC at earlier ages and in more aggressive forms, with a higher prevalence of metastasis. Genetic studies, including those focused on *BRCA1* and *BRCA2* genes, have revealed population-specific variations in BC susceptibility. Despite efforts to investigate BC genetics in African and African-descendant populations, research remains limited compared to studies conducted in populations of European descent. Socioeconomic factors further compound the challenges faced by marginalized populations, influencing disease outcomes and treatment efficacy. This review explores the BC literature in African and African-descendant populations, highlighting population-specific genetic variants associated with the disease’s subtypes, treatment response, and disease evolution. Limited sample sizes and lack of data on genetic ancestry hinder the development of precise risk stratification and treatment strategies. Efforts to expand research, improve data collection, and enhance genetic analyses in diverse populations are crucial steps toward addressing racial disparities and advancing BC care on a global scale.

## 1. Introduction

According to the Global Cancer Observatory (GLOBOCAN), it is estimated that approximately one in five individuals will be affected by breast cancer (BC) during their lifetime, and one in twelve women will die because of it. Female BC alone accounted for 2.3 million new cases in 2022, making it the second most common cancer type, with an incidence rate of 11.6%. Furthermore, it ranks as the fourth most lethal cancer, accounting for 6.9% of all cancer-related deaths (665,684 deaths).

BC is the most common cancer among women in 157 out of 185 countries and the most lethal cancer in 112 countries, with Melanesia and West Africa exhibiting the highest mortality age-standardized rates (ASRs) of 26.8 and 22.6 per 100,000 females, respectively [1]. Socioeconomic factors such as access to early diagnosis, personalized treatments, and quality of life significantly influence the burden of BC [2]. In the United States, there are also significant racial disparities. Black women have a 38% higher mortality rate despite a 5% lower incidence rate compared to White women. Black women are also more likely to develop high-grade tumors (38% compared to 24% in White women) [3]. Additionally, Black women have a higher likelihood of developing triple-negative breast cancer (TNBC) (19% versus 9–11% in other groups), lower survival rates (84% compared to 88–93% considering all stages) [3], and are more likely to develop the disease at an earlier age, before the age of 40 [4]. In most places, the recommended age for the first mammography is around 50 years old [5,6,7], an age at which most cases occur in White women without a family history of BC. However, this may be too late for early diagnosis in Black women. Within Africa, TNBC is more common in West Africa, particularly in Ghana (57% of the patients in the studies reviewed), Nigeria (49%), Senegal (47%), and Mali (46%) [8].

While the BC incidence rate in Africa is lower than in Europe and North America (40.5 per 100,000 individuals per year in Africa compared to 75.6 in Europe and 95.1 in North America), the mortality rate is significantly higher (19.2 per 100,000 individuals per year in Africa compared to 14.6 in Europe and 12.3 in North America) [9]. Within Africa, incidence rates also vary significantly. Northern (53.2 per 100,000 women in 2022) and Southern Africa (46.2) have the highest rates, while Western (42.1), Eastern (31.9), and Middle Africa (26.7) present considerably lower rates [1]. However, Western Africa exhibits the highest BC mortality rate within the continent (22.6 per 100,000 individuals per year) [1]. A significant contributor to the high mortality rates is late diagnosis, often due to long distances to healthcare facilities, inadequate healthcare infrastructure, and limited access to screening programs. These factors not only impact mortality rates but also pose significant challenges to accurately estimating BC incidence due to the underreporting of cases [10,11,12].

Breast cancer also imposes a substantial economic burden globally. The overall economic cost of all cancers between 2020 and 2050 is projected to be around USD 25.2 trillion. BC alone accounts for USD 2 trillion, with Brazil and Algeria bearing the highest macroeconomic costs [13]. The costs of BC diagnosis and treatment vary considerably, with higher costs in countries with higher per capita income [13]. In contrast, middle- and low-income countries face productivity losses due to high mortality and morbidity rates. Treatment costs significantly increase with advanced disease stages and poorer prognosis [13,14,15]. Therefore, early detection and timely access to effective treatment are crucial not only for improving patient outcomes but also for mitigating the economic burden of BC.

In the context of BC genetics, the most strongly associated genetic variants are found in the *BRCA1* and *BRCA2* genes, the majority of which are associated with hereditary BC and the TNBC subtype [16,17,18,19]. Interestingly, some African populations, including Algerian, Moroccan, Nigerian, and Egyptian, present higher frequencies of *BRCA1* and *BRCA2* mutations compared to the global average [16,20,21,22]. These findings highlight the importance of considering population-specific genetic factors in BC risk assessment and management.

A comprehensive review by Abbad et al. (2018) [23] analyzed 43 case-control studies investigating genetic factors associated with BC in African populations. Only 17% of African countries have conducted research on BC genetics, most solely focusing on *BRCA1/2* mutations. Notably, the mutation (c.798_799delTT) was exclusively found in Northern Africa. In addition to *BRCA* mutations, variants in genes such as *HER2*, *TP53*, *APOBEC3*, and *MTHFR* were also identified in Northern Africa. A more recent review, conducted by Hayat et al. (2021) [24], found relevant data on only 22% of Sub-Saharan African countries. They highlighted six genes with mutations that were predicted to be pathogenic for BC: *BRCA1*, *BRCA2*, *PALB2*, *ATM*, *BARD1*, and *RAD51C*.

In contrast, research conducted outside of the African continent more often uses advanced techniques, such as multiple gene prognostic assays. These approaches provide valuable information for personalized treatment by identifying potential biomarkers predicting treatment response.

Since the review conducted by Abbad et al. in 2018 [23] and the most recent study conducted by Hayat in 2021, which showed a lack of molecular studies in Sub-Saharan Africa despite the increasing incidence of BC in these regions [24], significant advances have been made in BC research. Therefore, this review aims to synthesize recent findings in African and African diaspora populations, focusing on identifying novel genetic variants associated with BC susceptibility and characterizing disease subtypes of higher prevalence in African descendants. In summary, we aimed to present the most recent findings that contribute to the state of the art in African population-based BC research. This allows us to identify knowledge gaps in BC genetic research among Black women, a crucial step in the development of more effective and equitable BC-care strategies for women of African descent.

## 2. Materials and Methods

### 2.1. Search Strategy and Eligibility Criteria

A comprehensive literature search was conducted using the PubMed and Web of Science databases to identify studies published on BC in populations of African ancestry. This search yielded 986 articles. To refine the search, we included only studies specifically focused on African and African-descendant populations. Priority was given to studies investigating the two most aggressive BC subtypes: TNBC and HER2-positive, which have been shown to have a higher prevalence among African-descendant populations [17,25,26,27,28]. Additionally, studies that explicitly reported the self-identified ancestry of participants were prioritized to ensure the accurate representation of African ancestry within the study cohorts. Following this initial search, the GWASs catalog was consulted to identify genome-wide association studies (GWASs) in African or African-descendant populations.

### 2.2. Study Selection

**Figure 1:**
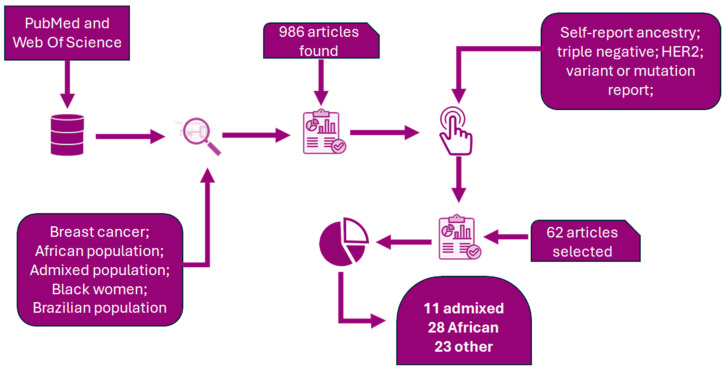
Flowchart of the literature search and selection process.

This approach resulted in the selection of 60 articles for inclusion in this narrative review. A visual summary of the research and selection criteria is presented in Figure 1, and the complete list of the included articles is provided in Supplementary Table S1.

## 3. Results

### 3.1. Breast Cancer Genetics

Breast cancer is a complex disease with diverse classifications. While only 5–10% of cases are hereditary, primarily linked to germline mutations in the *BRCA1* and *BRCA2* genes, the majority are sporadic, caused by the accumulation of somatic mutations over time, leading to uncontrolled cell growth and tumor development [1,2,29,30].

Molecular subtypes of BC are identified based on the expression of estrogen receptor (ER), progesterone receptor (PR), human epidermal growth factor receptor 2 (HER2), and the concentration of the Ki-67 antigen, a marker of cell proliferation [31,32,33]. These biomarkers provide valuable prognostic and predictive information, guiding treatment decisions and influencing patient outcomes [2,32]. A summary of BC subtypes is presented in Table 1, and a review of the classification can be found in Zhang 2023 [34].

Luminal A and Luminal B subtypes have the best prognosis, with Luminal A being less aggressive due to a lower proliferation rate. Due to its higher proliferation rate, Luminal B is considered to be intermediate/advanced grade. HER2-positive subtypes generally have a poorer prognosis compared to other subtypes. The HER2-enriched subtype (characterized by a Ki67 rate exceeding 30% and the absence of hormone receptors) is more aggressive than the Luminal HER2 subtype. The triple-negative BC (TNBC), lacking hormone receptors and HER2, exhibits high proliferative activity and is more prevalent in Black women, patients under 40 years old, and in advanced stages of the disease [2,32,35].

The subtype TNBC comprises 15–20% of BC cases and has a low survival rate due to late diagnosis and a high incidence of metastasis (mainly in the liver and lungs, in addition to bones and the central nervous system). The TNBC is divided into six types based on gene expression profiles and ontology: immunomodulatory, luminal androgen receptor, mesenchymal stem-like, mesenchymal, basal-like 1, and basal-like 2. About 80% of TNBC is basal-like, characterized by high expression of DNA damage response and cell cycle-related genes, often associated with chemotherapy sensitivity. Targeting therapies, particularly for patients with BRCA mutations, include PARP inhibitors and immune checkpoint inhibitors that inhibit the binding of PD1 and PD-L1, decrease T cell exhaustion, and, therefore, strengthen the antitumor response [36].

In addition to subtype evaluation, it is necessary to identify BC grade and stage. The worst prognoses and highest metastatic capacity are observed in HER2-positive and TNBC subtypes, typically found in stages 3 or 4 of the disease, where cancer cells have spread to axillary lymph nodes or other organs, and tumors are 5 cm or larger [2,25,37].

Advances in technology are enabling the identification of genetic alterations specific to each subtype, with researchers identifying variants linked to subtypes and even specific ethnicities [37,38]. High penetrance BC genetic variants include *BRCA1* and *BRCA2* mutations. Moderate penetrance variants are often involved in DNA repair mechanisms, and low penetrance variants require a combination of multiple individual variants to increase disease risk [39].

Hamdi et al. (2016) [40] investigated 313 eQTLs (expression Quantitative Trait Loci) in 175 genes involved in homologous recombination, interstrand crosslink DNA repair, and the modulation of cellular functions of *BRCA1* and *BRCA2*, among other characteristics related to BC susceptibility. Three SNPs had significant associations with BC. The main one was rs11099601, a 3′ UTR variant of the *ABRAXAS1* (*FAM175A*) gene, on chromosome 4, identified as a novel risk factor associated with ER-positive BC. This variant, although not present in *BRCA1* and *BRCA2* mutation carrier patients, was found together with mutations in *MRPS18C* and *HELQ*. *MRPS18C* belongs to the Mitochondrial Ribosomal Protein family, and its upregulation is a predictive marker for unfavorable BC survival outcomes [41]. *HELQ* was suspected to be a predisposition gene to breast and ovarian cancer but was not associated with BC in the Finnish population [42]. The study [40] was performed with individuals of European ancestry, so the findings may not apply to other ethnicities. For example, the associated allele of rs11099601 had a 50% frequency, but in African populations, it had a frequency of 9–24%, as reported by Ensembl (1000 Genomes project’s samples). In addition, linkage disequilibrium with other SNPs is weaker in African populations, and in this case, no SNP presented a perfect correlation with rs11099601. Indeed, BC’s genetic profile can be different depending on the individual’s ancestry. One example is the occurrence of inactivating *FBXW7* mutations, a critical tumor suppressor gene, observed in African Americans but not in European American or Kenyan patients [43].

Unfortunately, there is a huge problem of underrepresentation of marginalized populations in genetic and biomedical research, which significantly hinders the development of precision medicine applications [44]. Initiatives like the African Organisation for Research and Training in Cancer (AORTIC) and the African Research Group for Oncology (ARGO) are working to increase research participation among African populations. The African American Breast Cancer Epidemiology and Risk (AMBER) Consortium, together with the African American Breast Cancer Consortium, recently formed the Breast Cancer Genetic Study in African Ancestry Populations initiative to study more than twenty thousand Black women with BC. The African Ancestry Breast Cancer Genetic (AABCG) consortium recently published the largest GWAS of BC in Black women, with more than forty thousand patients [45], which will be discussed in the following sections of this review.

Admixed populations of the African diaspora, such as Latin Americans [46,47], are also severely underrepresented in genomics research. However, investigating their genetic composition can help to understand the impact of different population ancestries on the variation in disease susceptibility [48]. Fortunately, a significant international effort is underway to investigate BC, the US–Latin America Cancer Research Network, which focuses on studying molecular subtypes of BC in premenopausal women in Latin America [49]. Unfortunately, most of the studies in Latin America still exhibit a bias toward White patients, with databases enriched for European ancestry.

### 3.2. Breast Cancer Genetic Studies in African and African Ancestry Populations

Black women with BC have a worse prognosis and higher mortality [50], but the reasons for this discrepancy are still not completely understood [51]. Socioeconomic factors are certainly an important part of the problem, but studies have shown that even when adjusted for socioeconomic factors, Black women are still at a higher risk, which suggests the involvement of genetic factors [26]. Despite these disparities, fewer studies are conducted in African-descendant populations [26,51].

Two BC subtypes, HER2+ and TNBC (characterized by the absence of hormone receptors and HER2), have a higher incidence in women with African ancestry [25,26]. These subtypes have the worst prognosis among BC subtypes, necessitating various concurrent therapies [2,3]. Another BC subtype that occurs more frequently in Black women than in White women is inflammatory BC. This histological subtype is characterized by aggressive behavior and rapid progression, classified as a stage IIIB, IIIC, or IV depending on evidence of metastasis. Common physical changes in the breast include warmth, erythema, and edema (called peau d’orange), usually without a well-defined mass, and a hallmark of inflammatory BC, the presence of emboli within the dermal lymphatic system [52,53]. In Tunisia, Egypt, and Morocco, there is a high rate of inflammatory BC due to overexpression of the RhoCGTPase protein. This protein modulates angiogenesis genes, cellular adhesion, and invasion, leading to a higher incidence of metastasis [22,50,52,53,54,55,56,57].

**Figure 2:**
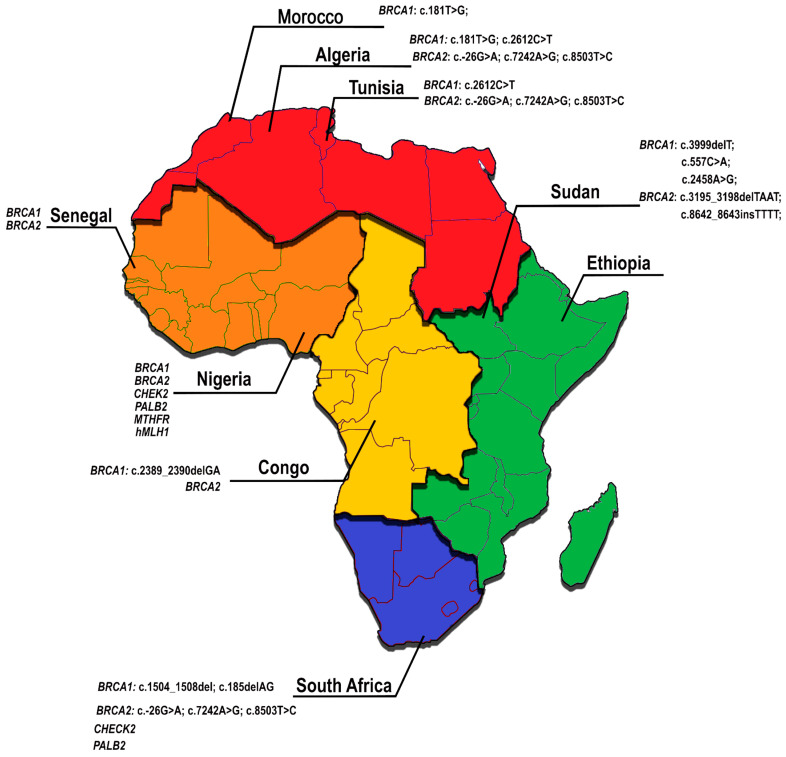
Most relevant genes and mutations found in each African country that were associated with BC susceptibility and/or subtypes. Based on Abbad et al. (2018) [23].

Abbad et al. (2018) [23] summarized the main genetic variants found in genes known to be related to BC in different regions of the African continent (some of these findings can be seen in Figure 2). They highlighted the significant discrepancy between these studies and those conducted in other regions and emphasized the need for more advanced analyses, larger sample sizes, and improved financial capacity to support better prognostic examinations [26]. A study that had a slightly larger sample size was published by Gaceb et al. (2018) [17]. They found a high proportion of the TNBC subtype in Algerian women with a familial history of BC by identifying a high frequency of *BRCA1* mutations.

A high frequency of deleterious *BRCA1/BRCA2* mutations was reported in Algerian patients below the age of 40 (8 out of 40 patients, each with a distinct mutation) [22]. Notably, one was a novel germline *BRCA2* mutation (c.6450del). A significant proportion of those patients (7 out of 8) were from the urban Kabyle population, and the authors suggested that their urban, sedentary lifestyle might modify the penetrance of the mutations. Interestingly, all *BRCA1* carriers presented TNBC, while the four *BRCA2* carriers were all positive for hormonal receptors (with one also positive for HER2). Most patients in the study were treated with mastectomy due to the lack of access to radiotherapy centers [22].

A BC GWAS with women of African ancestry identified an African-specific variant, rs9833271, as a risk factor for estrogen-negative BC [38]. This intergenic variant is located 37.3 kb upstream of the *TNFSF10* gene on chromosome 3. Tumor Necrosis Factor Superfamily Member 10 (TNFSF10), also known as TRAIL or APO2L, is a crucial mediator of p53-dependent apoptosis [58]. The absence of TNFSF10 receptors has been linked to increased tumor incidence across various contexts [58]. Therefore, in the study, the authors suggested the *TNFSF10* gene as a plausible causal gene for the association, attributed to the heightened sensitivity of TNBC cells to TNFSF10-induced apoptosis. Notably, this variant is a second association signal within this genomic region, while the first association was not specific to African patients.

Martini et al. (2022) [37] demonstrated that genetic ancestry significantly influences TNBC tumor biology even within the African continent (comparison between Ethiopian, Ghanaian, and African American patients). The study shows that in populations of African ancestry, the higher prevalence of TNBC is attributed to distinct immune response pathways compared to those observed in patients of European descent. Furthermore, they emphasize the critical need for the inclusion of multi-ethnic samples in genomic research to enhance both the rigor of research and its potential to reduce racial disparities in healthcare outcomes. A key point raised by the authors is the inadequacy of simplistic approaches to ancestry inferences, highlighting the need for deep genomic analysis that can identify subcontinental ancestry.

African genomic ancestry was also associated with BC in Brazilian women. Continental ancestry was inferred using 46 ancestry informative markers (AIMs), and African ancestry was associated with a higher susceptibility to the HER2+ and TNBC subtypes, as well as poorer prognosis [25]. Furthermore, TNBC was more frequent in young women (below the age of 40), similar to observations in African Americans. Intriguingly, mortality rates in young women are increasing in Brazil [59], particularly in the Northeast region, where the study found the highest proportions of African ancestry.

### 3.3. Breast Cancer Genomic Studies in African and African Ancestry Populations

Since 2007, the GWAS approach has been employed to investigate the genetic influences on BC in large cohorts, ranging from thousands to tens of thousands of patients [60]. This led to the identification of approximately 200 loci [60] and more than 2000 genetic variants associated with BC or related traits (according to the GWASs catalog). However, the first GWAS to include a substantial number of BC patients of African ancestry was published in 2011, encompassing 3749 African American women (1004 cases) [61]. This study identified the *TERT-CLPTM1L* locus on chromosome 5p15 as associated with TNBC and HER2-negative BC, which are more prevalent in Black women. Subsequently, in 2012, a GWAS specifically focused on African American BC patients was published (n = 5984, of which 3153 cases were in the main cohort, and replication testing of the top 66 associations was carried out in an additional 3607 BC cases and 11,330 controls). This study identified novel loci associated with BC, with two associations replicated in the second cohort, rs4322600 (an intergenic variant 100 kb upstream of the *GALC* gene at 14q31) and rs10510333 (another intergenic variant, 486 kb upstream of the *GRM7* gene at 3p26) [62].

Since the first GWAS [45] with a replication sample including African American BC patients (n = 870), it became evident that the results might not be universally applicable across all populations. The initial study, conducted in a European cohort, identified two SNPs (rs13387042 and rs3803662) associated with ER-positive BC. Neither association was replicated in the African American cohort. Actually, one of the susceptibility alleles identified in the European cohort (rs3803662*T) was a protective allele in the African American sample (the other SNP showed no significant association). This discrepancy was attributed to potential differences in linkage disequilibrium patterns between the investigated allele and the causal allele. While this is a plausible explanation, the reasons for the differing associations may be more complex, potentially involving gene–gene interactions that result in distinct functional impacts. Regardless of the specific mechanisms, the crucial takeaway is the imperative need to investigate each population independently to fully understand the genetic factors influencing disease susceptibility.

In a search for causal variants, a cross-ancestry GWAS meta-analysis identified risk loci with consistent direction of associations in African and European descendants [63]. Four loci were associated with BC risk (1p13.3, 5q31.1, two independent signals at 15q24, and another at 15q26.3). Two additional loci were associated with ER-negative BC (1q41 and 7q11.23). The identified SNPs were noncoding, with four located in introns (genes *KCNK2* -rs67931591, at 1q41-, *C5orf56* -rs2522057, at 5q31.1-, *SCAMP2* -rs1869952, at 15q24.1-, and *SIN3A* -rs60381548, at 15q24.2-) and the others intergenic (rs17024629, at 1p13.3, rs1637365, at 7q11.23, and rs181337095, at 15q26.3). Some of these SNPs were eQTLs of their own gene or of other genes, and the authors discussed their possible impact on gene expression. The intergenic rs17024629*T allele, at 1p13.3, was associated with increased expression of the carcinogen metabolism genes *GSTM1*, *GSTM2*, and *GSTM4* (19, 31, and 51 kb downstream). The intergenic SNP rs1637365, at 7q11.23, was associated with the expression of the pseudogene *STAG3L2*. The rs2522057, located in an intron of *C5orf56*, was associated with *IRF1* gene expression (a gene with BC tumor suppressor functions) and histone modifications. This SNP was also associated with *SLC22A5* gene expression in normal breast tissue of the GTEx project. The rs1869959, located in an intron of the *SCAMP2* gene, was associated with gene expression of the *ULK3* and the *MPI* gene (12 and 35 kb upstream). The association with *ULK3* was also reported by GTEx in normal breast tissue. Lastly, the rs60381548 intronic SNP of the *SIN3A* gene (Switch-independent 3 family A, a transcriptional regulator previously implicated in BC progression and response to chemotherapy) was associated with its own gene expression, as well as the expression of *PTPN9*, *SNUPN*, and *SNX33* (30 and 162 kb downstream and 212 kb upstream, respectively). This SNP was also associated with histone modifications.

To date, of the 145 GWASs investigating BC, only 19 (13%) included African or African ancestry patients in the main cohort (Supplementary Table S2) according to the GWASs catalog. Three additional GWASs included African and African ancestry patients in their replication cohorts [45,64,65]. Furthermore, of the nineteen GWASs that involved African or African ancestry populations, only seven were conducted in African patients, while the remaining twelve included only African American or African Caribbean BC patients. The studies in the African continent included a very limited number of African countries. The majority of studies included participants from Ghana and Nigeria, while one study also included participants from Uganda and Cameroon. Invariably, the sample sizes of African and African ancestry patients were considerably smaller than those of other ancestries (typically tens or hundreds of individuals).

Another significant limitation of current BC GWASs lies in the predominant use of genotyping arrays. With a few exceptions, these arrays were primarily developed based on European populations, exhibiting inherent biases when applied to diverse populations, particularly those of African ancestry. This is due to differences in allele frequencies and linkage disequilibrium patterns between populations, which can limit the identification of associated variants. Moreover, of the four GWASs that utilized sequencing technologies (whole-genome or exome sequencing), only one included patients of African ancestry.

The largest cohort of BC patients of African ancestry in a GWAS was published in 2024 [60] by the African Ancestry Breast Cancer Genetics (AABCG) Consortium. It included 40,138 women (18,034 cases), of which 85% were from the United States and 15% from the African continent and Barbados, significantly enhancing diversity in BC research. They identified 12 loci associated with BC risk, summarized in Table S3. Among these, a novel associated SNP, rs76664032 (an intergenic variant 10 kb from the 3′-UTR of the long noncoding RNA gene *RP11-19E11.1* at 2q14.2, for which the SNP is an eQTL), was strongly associated with TNBC with an odds ratio (OR) of 1.30. The risk allele (A) frequency was 81% in populations of African ancestry (substantially lower than the >99% observed in European ancestry populations). The authors highlight that this gene is a target of E2F1, a transcription factor involved in cell proliferation, and is upregulated in basal-like BC. However, the risk allele was associated with lower expression levels of the lncRNA. Two other strong associations (ORs of 1.38) were observed for TNBC, replicating weaker associations previously found in populations of European ancestry (OR = 1.26 and 1.21). The variants were rs10069690 (an intronic variant within the *TERT* gene (intron 4) at 5p15.33) and rs12974508 (an intergenic variant 1.4 kb from the *ABHD8* gene and 1.9 kb from the *MRPL34* gene at 19p13.11). Both risk alleles presented higher frequencies in African ancestry populations (61% and 60%, respectively) compared to European ancestry populations (26% and 48%, respectively). Additionally, rs10853615 (an intronic variant within the *TTC39C* gene (intron 1) at 18q11.2) was a novel locus associated with ER+ BC (OR: 1.15). The authors mentioned that this gene has been previously reported to be upregulated in cell lines with loss-of-function mutations in *STK11*, a major tumor suppressor gene in lung cancer. The study also identified a novel variant strongly associated with overall BC risk (OR = 1.48), rs61751053 (a missense variant (p.Ser148Leu) within the *ARHGEF38* gene). The authors mentioned that this gene belongs to the Rho family of GTPase regulators and has been previously implicated as a potential biomarker for both aggressive prostate cancer and lung cancer. The risk allele (T) is of low frequency (1–2%) in populations of African ancestry and rare in European and Asian ancestry populations (>0.1%). These findings underscore the critical importance of conducting research in diverse populations and the need for larger sample sizes.

## 4. Discussion and Conclusions

To date, the majority of BC studies have been conducted on European or European-descendant women, overlooking a significant portion of human genetic diversity, which is predominantly observed in African populations. Recognizing the substantial genetic diversity both within and between African populations is essential for medical genomics and health studies, as it is crucial for understanding the impact of genetic variants on disease susceptibility or protection. Even when focusing on the African continent, it is important to acknowledge the diversity among its populations. Most of the studies reviewed included countries from the same African regions, such as Nigeria, Ghana, Senegal, and Algeria.

Regarding the African diaspora populations, such as those in Brazil (the country that has the largest population of the African diaspora), the primary genetic contributions originate from West African populations, including those from Niger, Ghana, Nigeria, and Senegal, who were transported to the North and Northeast regions of Brazil, particularly Bahia and Pernambuco. Additionally, contributions arise from East African populations, such as those from Mozambique, Ethiopia, and Kenya, who were mainly brought to the South and Southeast regions of Brazil [48]. This migration history provides valuable insights into the potential genetic variations present within the African diaspora population.

Numerous studies have identified a higher incidence of TNBC and HER2+ BC in women of African descent. However, our current knowledge is insufficient to effectively translate this information into personalized medicine and improve treatment outcomes for these women. For example, the Brazilian public health system, with its motto of “health for all”, does not achieve equitable healthcare access for Black women. There is a critical need for more accurate data, including information on ancestry, to address these disparities.

As noted in Abbad’s research, a substantial gap persists in cancer and BC research, particularly within African countries. The costs associated with diagnosing and treating BC are substantial, ranking as the third highest among all cancers. These costs are projected to have a significant financial impact on countries over the next 30 years. In developing countries, these costs are primarily driven by high mortality rates associated with the disease. The most effective strategy to mitigate these costs involves a two-pronged approach: investing in research focused on local populations, which includes studies that investigate the unique genetic, environmental, and socioeconomic factors that influence BC risk and outcomes within specific African contexts, and implementing public health policies that ensure more accurate and timely diagnosis for vulnerable groups, which includes improving access to screening programs, early detection services, and affordable treatment options for all individuals.

One of the common challenges encountered across studies is the limitation of sample size, which can significantly impact the reliability and accuracy of findings, particularly within highly diverse populations. The ability to analyze an individual’s local chromosomal ancestry emerges as a valuable tool, enabling greater precision in personalized medicine and unlocking the potential for identifying novel treatment targets and detecting hidden variants in admixed populations.

Studies such as the GWAS conducted by Jia et al. [60], which significantly increased our understanding of genetic variants correlated with BC in African populations, contribute to opening new avenues for research and therapeutic options within this population. One important application is the calculation of polygenic risk scores (PRSs), which are often developed for European populations and exhibit lower accuracy in African populations [66]. The associations identified by Jia and collaborators [64] yielded a PRS with an area under the curve (AUC) of 0.60 for predicting BC risk in African American women, outperforming previous PRSs derived from studies in populations of European ancestry. These findings highlight the critical need for the inclusion of diverse populations in genetic studies to refine risk prediction and improve personalized prevention strategies [44,45,54,67,68]. Even for populations of European ancestry, the routine clinical use of PRS in sporadic BC risk assessment is still under investigation and not yet widely recommended.

On the other hand, risk assessment for hereditary BC can be conducted using a variety of methods, typically involving a combination of personal and family history factors. These assessments may also incorporate genetic information such as *BRCA1* and *BRCA2* mutations. Risk assessment can provide personalized recommendations that may benefit patients by aiding in the determination of eligibility for risk-reducing interventions, including medication or surgical options, screening modalities, the age at which to initiate breast screening, and the frequency of these examinations [69]. However, the accuracy of these risk assessment methods may vary across different populations and can increase disparities if applied without caution. Examples include the Tyrer–Cuzick (IBIS) [70], CanRisk [71,72], and polygenic risk scores algorithms [73,74,75], which were created based on incidence and prevalence data for women in European cohorts. As discussed in this review, genetic variants associated with BC and their impact often differ across populations of different ancestries. Therefore, the selection of variants for testing should consider the patient’s population or ancestry. A recent study investigated the specificities of BC risk among women of different ancestries in South Africa [76]. A lower proportion of Black patients reported a family history of BC, which could be attributed to a lower incidence, lower awareness of family history, or lower penetrance of the mutations. Authors therefore recommend that women with BC who have any family history of hereditary BC-associated cancers should be offered genetic testing even if they do not meet the defined criteria. Another example of a potentially problematic approach is the use of age at diagnosis of family members as a risk factor. Early-onset BC is often defined as occurring before the age of 50. However, Black women experience a higher incidence of sporadic BC at younger ages (between 40 and 50 years old). Consequently, age at diagnosis may not be an equally reliable indicator of familial BC risk in Black women, necessitating careful consideration of this factor in conjunction with other genetic and demographic factors.

Even without methods including genetic data from patients of African ancestry, women with a family history of BC can still benefit from cancer genetic counseling. However, medical referral rates for genetic counseling are lower for Black BC patients compared to White patients, highlighting another significant barrier to accessing care [77]. Limited access to clinics, demographic and socioeconomic disparities, and lack of knowledge of family history can hinder the establishment of equitable cancer risk assessment services. An alternative approach could involve integrating individualized risk assessment as a component of primary care follow-up for medically underserved women. This approach resulted in a 51% uptake of mammography screening compared to 37% among women receiving usual care [78].

Our review highlights three main points: there is a gradual, though modest, increase in studies on BC in African and African diaspora populations; however, these studies are often limited by sample size compared to those conducted in European populations, and they will likely require comparatively larger sample sizes due to the higher genetic diversity within these populations; there is a critical need to supplement national and international databases with research involving African ancestry populations.

While this review does not strictly adhere to the methodology of a systematic review—as it included a pre-existing review and also incorporated a search on the GWASs catalog—a research protocol was established (outlined in the Methods section). This protocol aimed to include all articles investigating the genetics of breast cancer in African populations or populations of the African diaspora. The search methodology included various databases and predefined keywords and established clear inclusion and exclusion criteria, with titles and abstracts reviewed for eligibility. We believe that this approach enhances this review by providing valuable information without compromising its overall quality.

## Figures and Tables

**Table 1:** Breast cancer subtypes based on immunohistochemical profiling.

BC Subtype	ER Status	PR Status	HER2 Status	Ki67 Level
Luminal A	Positive	Positive/Negative	Negative	Low
Luminal B	Positive	Positive/Negative	Positive/Negative	High
HER2	Negative	Negative	Positive	High
TNBC	Negative	Negative	Negative	High

BC: breast cancer; TNBC: triple-negative breast cancer; ER: estrogen receptor; PR: progesterone receptor; HER2: human epidermal growth factor receptor 2; Ki67 high: >14%; Ki67 low: <14% [15,32].

## Data Availability

All relevant data are included within the article and/or the Supplementary Materials.

## Supplementary Materials

The following supporting information can be downloaded at https://www.mdpi.com/article/10.3390/genes16020199/s1, Table S1: Summary of articles used in our research; Table S2: Summary of BC GWASs with African or African ancestry populations; Table S3: Summary of the most relevant SNPs found on Jia G. GWAS [62].

## References

[1] Bray F., Laversanne M., Sung H., Ferlay J., Siegel R.L., Soerjomataram I., Jemal A. (2024). Global Cancer Statistics 2022: GLOBOCAN Estimates of Incidence and Mortality Worldwide for 36 Cancers in 185 Countries. CA A Cancer J. Clin..

[2] Harbeck N., Penault-Llorca F., Cortes J., Gnant M., Houssami N., Poortmans P., Ruddy K., Tsang J., Cardoso F. (2019). Breast Cancer. Nat. Rev. Dis. Primers.

[3] Giaquinto A.N., Miller K.D., Tossas K.Y., Winn R.A., Jemal A., Siegel R.L. (2022). Cancer Statistics for African American/Black People 2022. CA A Cancer J. Clin..

[4] Chen T., Kharazmi E., Fallah M. (2023). Race and Ethnicity–Adjusted Age Recommendation for Initiating Breast Cancer Screening. JAMA Netw. Open.

[5] INCA Câncer de Mama: Vamos Falar Sobre Isso?. https://www.inca.gov.br/publicacoes/cartilhas/cancer-de-mama-vamos-falar-sobre-isso.

[6] Migowski A., Dias M.B.K., Nadanovsky P., Silva G.A.E., Sant’Ana D.R., Stein A.T. (2018). Diretrizes para detecção precoce do câncer de mama no Brasil. III—Desafios à implementação. Cad. Saúde Pública.

[7] Migowski A., Corrêa F.d.M. (2020). Recomendações para detecção precoce de câncer durante a pandemia de covid-19 em 2021. Rev. De APS.

[8] Hercules S.M., Alnajar M., Chen C., Mladjenovic S.M., Shipeolu B.A., Perkovic O., Pond G.R., Mbuagbaw L., Blenman K.R., Daniel J.M. (2022). Triple-Negative Breast Cancer Prevalence in Africa: A Systematic Review and Meta-Analysis. BMJ Open.

[9] Liao L. (2024). Inequality in Breast Cancer: Global Statistics from 2022 to 2050. Breast.

[10] Azubuike S.O., Muirhead C., Hayes L., McNally R. (2018). Rising Global Burden of Breast Cancer: The Case of Sub-Saharan Africa (with Emphasis on Nigeria) and Implications for Regional Development: A Review. World J. Surg. Onc..

[11] Adeoye P.A. (2023). Epidemiology of Breast Cancer in Sub-Saharan Africa. Breast Cancer Updates.

[12] Hamdi Y., Abdeljaoued-Tej I., Zatchi A.A., Abdelhak S., Boubaker S., Brown J.S., Benkahla A. (2021). Cancer in Africa: The Untold Story. Front. Oncol..

[13] Chen S., Cao Z., Prettner K., Kuhn M., Yang J., Jiao L., Wang Z., Li W., Geldsetzer P., Bärnighausen T. (2023). Estimates and Projections of the Global Economic Cost of 29 Cancers in 204 Countries and Territories From 2020 to 2050. JAMA Oncol..

[14] Blumen H., Fitch K., Polkus V. (2016). Comparison of Treatment Costs for Breast Cancer, by Tumor Stage and Type of Service. Am. Health Drug Benefits.

[15] Sun L., Legood R., dos-Santos-Silva I., Gaiha S.M., Sadique Z. (2018). Global Treatment Costs of Breast Cancer by Stage: A Systematic Review. PLoS ONE.

[16] Dobbin E.A.F., Medeiros J.A.G., Costa M.S.C.R., Rodrigues J.C.G., Guerreiro J.F., Kroll J.E., Souza S.J.D., de Assumpção P.P., Ribeiro-dos-Santos Â., Santos S.E.B.D. (2021). Identification of Variants (Rs11571707, Rs144848, and Rs11571769) in the BRCA2 Gene Associated with Hereditary Breast Cancer in Indigenous Populations of the Brazilian Amazon. Genes.

[17] Gaceb H., Cherbal F., Bakour R., Ould-Rouis A., Mahfouf H. (2018). Clinicopathological and Molecular Study of Triple-Negative Breast Cancer in Algerian Patients. Pathol. Oncol. Res..

[18] Silva F.C., Lisboa B.C., Figueiredo M.C., Torrezan G.T., Santos É.M., Krepischi A.C., Rossi B.M., Achatz M.I., Carraro D.M. (2014). Hereditary Breast and Ovarian Cancer: Assessment of Point Mutations and Copy Number Variations in Brazilian Patients. BMC Med. Genet..

[19] Van der Merwe N.C., Combrink H.M., Ntaita K.S., Oosthuizen J. (2022). Prevalence of Clinically Relevant Germline BRCA Variants in a Large Unselected South African Breast and Ovarian Cancer Cohort: A Public Sector Experience. Front. Genet..

[20] Bertwistle D., Swift S., Marston N.J., Jackson L.E., Crossland S., Crompton M.R., Marshall C.J., Ashworth A. (1997). Nuclear Location and Cell Cycle Regulation of the BRCA2 Protein. Cancer Res..

[21] Chen Z., Guo X., Long J., Ping J., Li B., Fadden M.K., Ahearn T.U., Stram D.O., Shu X.-O., Jia G. (2021). Discovery of Structural Deletions in Breast Cancer Predisposition Genes Using Whole Genome Sequencing Data from >2000 Women of African-Ancestry. Hum. Genet..

[22] Henouda S., Bensalem A., Reggad R., Serrar N., Rouabah L., Pujol P. (2016). Contribution of BRCA1 and BRCA2 Germline Mutations to Early Algerian Breast Cancer. Dis. Markers.

[23] Abbad A., Baba H., Dehbi H., Elmessaoudi-Idrissi M., Elyazghi Z., Abidi O., Radouani F. (2018). Genetics of Breast Cancer in African Populations: A Literature Review. Glob. Health Epidemiol..

[24] Hayat M., Chen W.C., Brandenburg J.-T., Babb de Villiers C., Ramsay M., Mathew C.G. (2021). Genetic Susceptibility to Breast Cancer in Sub-Saharan African Populations. JCO Glob. Oncol..

[25] da Costa Vieira R.A., Sant’Anna D., Laus A.C., Bacchi C.E., Silva R.J.C., de Oliveira-Junior I., da Silva V.D., Pereira R., Reis R.M. (2023). Genetic Ancestry of 1127 Brazilian Breast Cancer Patients and Its Correlation With Molecular Subtype and Geographic Region. Clin. Breast Cancer.

[26] Jiagge E., Chitale D., Newman L.A. (2018). Triple-Negative Breast Cancer, Stem Cells, and African Ancestry. Am. J. Pathol..

[27] Omilian A.R., Wei L., Hong C.-C., Bandera E.V., Liu S., Khoury T., Ambrosone C.B., Yao S. (2020). Somatic Mutations of Triple-Negative Breast Cancer: A Comparison between Black and White Women. Breast Cancer Res. Treat..

[28] Palmer J.R., Ruiz-Narvaez E.A., Rotimi C.N., Cupples L.A., Cozier Y.C., Adams-Campbell L.L., Rosenberg L. (2013). Genetic Susceptibility Loci for Subtypes of Breast Cancer in an African American Population. Cancer Epidemiol. Biomark. Prev..

[29] Kenemans P., Verstraeten R.A., Verheijen R.H.M. (2004). Oncogenic Pathways in Hereditary and Sporadic Breast Cancer. Maturitas.

[30] Van Der Groep P. (2006). Distinction between Hereditary and Sporadic Breast Cancer on the Basis of Clinicopathological Data. J. Clin. Pathol..

[31] Hassanpour S.H., Dehghani M. (2017). Review of Cancer from Perspective of Molecular. J. Cancer Res. Pract..

[32] Arzanova E., Mayrovitz H.N., Mayrovitz H.N. (2022). The Epidemiology of Breast Cancer. Breast Cancer.

[33] Orrantia-Borunda E., Anchondo-Nuñez P., Acuña-Aguilar L.E., Gómez-Valles F.O., Ramírez-Valdespino C.A., Mayrovitz H.N. (2022). Subtypes of Breast Cancer. Breast Cancer.

[34] Zhang X. (2022). Molecular Classification of Breast Cancer: Relevance and Challenges. Arch. Pathol. Lab. Med..

[35] Goldhirsch A., Wood W.C., Coates A.S., Gelber R.D., Thürlimann B., Senn H.-J. (2011). Strategies for Subtypes—Dealing with the Diversity of Breast Cancer: Highlights of the St Gallen International Expert Consensus on the Primary Therapy of Early Breast Cancer 2011. Ann. Oncol..

[36] Obidiro O., Battogtokh G., Akala E.O. (2023). Triple Negative Breast Cancer Treatment Options and Limitations: Future Outlook. Pharmaceutics.

[37] Martini R., Delpe P., Chu T.R., Arora K., Lord B., Verma A., Bedi D., Karanam B., Elhussin I., Chen Y. (2022). African Ancestry-Associated Gene Expression Profiles in Triple-Negative Breast Cancer Underlie Altered Tumor Biology and Clinical Outcome in Women of African Descent. Cancer Discov..

[38] Huo D., Feng Y., Haddad S., Zheng Y., Yao S., Han Y.-J., Ogundiran T.O., Adebamowo C., Ojengbede O., Falusi A.G. (2016). Genome-Wide Association Studies in Women of African Ancestry Identified 3q26.21 as a Novel Susceptibility Locus for Oestrogen Receptor Negative Breast Cancer. Hum. Mol. Genet..

[39] Mavaddat N., Antoniou A.C., Easton D.F., Garcia-Closas M. (2010). Genetic Susceptibility to Breast Cancer. Mol. Oncol..

[40] Hamdi Y., Soucy P., Adoue V., Michailidou K., Canisius S., Lemaçon A., Droit A., Andrulis I.L., Anton-Culver H., Arndt V. (2016). Association of Breast Cancer Risk with Genetic Variants Showing Differential Allelic Expression: Identification of a Novel Breast Cancer Susceptibility Locus at 4q21. Oncotarget.

[41] Lin X., Guo L., Lin X., Wang Y., Zhang G. (2022). Expression and Prognosis Analysis of Mitochondrial Ribosomal Protein Family in Breast Cancer. Sci. Rep..

[42] Pelttari L.M., Kinnunen L., Kiiski J.I., Khan S., Blomqvist C., Aittomäki K., Nevanlinna H. (2016). Screening of HELQ in Breast and Ovarian Cancer Families. Fam. Cancer.

[43] Tang W., Zhang F., Byun J.S., Dorsey T.H., Yfantis H.G., Ajao A., Liu H., Pichardo M.S., Pichardo C.M., Harris A.R. (2023). Population-Specific Mutation Patterns in Breast Tumors from African American, European American, and Kenyan Patients. Cancer Res. Commun..

[44] Aldrighetti C.M., Niemierko A., Van Allen E., Willers H., Kamran S.C. (2021). Racial and Ethnic Disparities Among Participants in Precision Oncology Clinical Studies. JAMA Netw. Open.

[45] Stacey S.N., Manolescu A., Sulem P., Rafnar T., Gudmundsson J., Gudjonsson S.A., Masson G., Jakobsdottir M., Thorlacius S., Helgason A. (2007). Common Variants on Chromosomes 2q35 and 16q12 Confer Susceptibility to Estrogen Receptor–Positive Breast Cancer. Nat. Genet..

[46] Adhikari K., Mendoza-Revilla J., Chacón-Duque J.C., Fuentes-Guajardo M., Ruiz-Linares A. (2016). Admixture in Latin America. Curr. Opin. Genet. Dev..

[47] Salzano F.M., Bortolini M.C. (2001). The Evolution and Genetics of Latin American Populations.

[48] Salzano F.M., Sans M. (2014). Interethnic Admixture and the Evolution of Latin American Populations. Genet. Mol. Biol..

[49] Zavala V.A., Serrano-Gomez S.J., Dutil J., Fejerman L. (2019). Genetic Epidemiology of Breast Cancer in Latin America. Genes.

[50] Lo A.-C., Kleer C.G., Banerjee M., Omar S., Khaled H., Eissa S., Hablas A., Douglas J.A., Alford S.H., Merajver S.D. (2008). Molecular Epidemiologic Features of Inflammatory Breast Cancer: A Comparison between Egyptian and US Patients. Breast Cancer Res. Treat..

[51] Silverstein A., Sood R., Costas-Chavarri A. (2016). Breast Cancer in Africa: Limitations and Opportunities for Application of Genomic Medicine. Int. J. Breast Cancer.

[52] Giordano S.H., Hortobagyi G.N. (2003). Inflammatory Breast Cancer: Clinical Progress and the Main Problems That Must Be Addressed. Breast Cancer Res..

[53] Robertson F.M., Bondy M., Yang W., Yamauchi H., Wiggins S., Kamrudin S., Krishnamurthy S., Le-Petross H., Bidaut L., Player A.N. (2010). Inflammatory Breast Cancer: The Disease, the Biology, the Treatment. CA Cancer J. Clin..

[54] Brignoni L., Cappetta M., Colistro V., Sans M., Artagaveytia N., Bonilla C., Bertoni B. (2020). Genomic Diversity in Sporadic Breast Cancer in a Latin American Population. Genes.

[55] Adedokun B., Zheng Y., Ndom P., Gakwaya A., Makumbi T., Zhou A.Y., Yoshimatsu T.F., Rodriguez A., Madduri R.K., Foster I.T. (2020). Prevalence of Inherited Mutations in Breast Cancer Predisposition Genes among Women in Uganda and Cameroon. Cancer Epidemiol. Biomark. Prev..

[56] Jiagge E., Jibril A.S., Chitale D., Bensenhaver J.M., Awuah B., Hoenerhoff M., Adjei E., Bekele M., Abebe E., Nathanson S.D. (2016). Comparative Analysis of Breast Cancer Phenotypes in African American, White American, and West Versus East African Patients: Correlation Between African Ancestry and Triple-Negative Breast Cancer. Ann. Surg. Oncol..

[57] Sastry K.S., Chouchane L., Ibrahim M.E., Rotimi C.N. (2019). Breast Cancer in African Populations. The Genetics of African Populations in Health and Disease.

[58] Kuribayashi K., Krigsfeld G., Wang W., Xu J., Mayes P.A., Dicker D.T., Wu G.S., El-Deiry W.S. (2008). TNFSF10 (TRAIL), a P53 Target Gene That Mediates P53-Dependent Cell Death. Cancer Biol. Ther..

[59] Silva J.D.D.E., de Oliveira R.R., da Silva M.T., Carvalho M.D.d.B., Pedroso R.B., Pelloso S.M. (2021). Breast Cancer Mortality in Young Women in Brazil. Front. Oncol..

[60] Jia G., Ping J., Guo X., Yang Y., Tao R., Li B., Ambs S., Barnard M.E., Chen Y., Garcia-Closas M. (2024). Genome-Wide Association Analyses of Breast Cancer in Women of African Ancestry Identify New Susceptibility Loci and Improve Risk Prediction. Nat. Genet..

[61] Haiman C.A., Chen G.K., Vachon C.M., Canzian F., Dunning A., Millikan R.C., Wang X., Ademuyiwa F., Ahmed S., Ambrosone C.B. (2011). A Common Variant at the TERT-CLPTM1L Locus Is Associated with Estrogen Receptor-Negative Breast Cancer. Nat. Genet..

[62] Chen F., Chen G.K., Stram D.O., Millikan R.C., Ambrosone C.B., John E.M., Bernstein L., Zheng W., Palmer J.R., Hu J.J. (2013). A Genome-Wide Association Study of Breast Cancer in Women of African Ancestry. Hum. Genet..

[63] Adedokun B., Du Z., Gao G., Ahearn T.U., Lunetta K.L., Zirpoli G., Figueroa J., John E.M., Bernstein L., Zheng W. (2021). Cross-Ancestry GWAS Meta-Analysis Identifies Six Breast Cancer Loci in African and European Ancestry Women. Nat. Commun..

[64] Hoffman J., Fejerman L., Hu D., Huntsman S., Li M., John E.M., Torres-Mejia G., Kushi L., Ding Y.C., Weitzel J. (2019). Identification of Novel Common Breast Cancer Risk Variants at the 6q25 Locus among Latinas. Breast Cancer Res..

[65] Tjader N.P., Beer A.J., Ramroop J., Tai M.-C., Ping J., Gandhi T., Dauch C., Neuhausen S.L., Ziv E., Sotelo N. (2024). Association of ESR1 Germline Variants with TP53 Somatic Variants in Breast Tumors in a Genome-Wide Study. Cancer Res. Commun..

[66] Gao G., Zhao F., Ahearn T.U., Lunetta K.L., Troester M.A., Du Z., Ogundiran T.O., Ojengbede O., Blot W., Nathanson K.L. (2022). Polygenic Risk Scores for Prediction of Breast Cancer Risk in Women of African Ancestry: A Cross-Ancestry Approach. Hum. Mol. Genet..

[67] Junior H.L.R., Novaes L.A.C., Datorre J.G., Moreno D.A., Reis R.M. (2022). Role of Polygenic Risk Score in Cancer Precision Medicine of Non-European Populations: A Systematic Review. Curr. Oncol..

[68] Hirko K.A., Rocque G., Reasor E., Taye A., Daly A., Cutress R.I., Copson E.R., Lee D.-W., Lee K.-H., Im S.-A. (2022). The Impact of Race and Ethnicity in Breast Cancer—Disparities and Implications for Precision Oncology. BMC Med..

[69] Hipp L.E., Hulswit B.B., Milliron K.J. (2022). Clinical Tools and Counseling Considerations for Breast Cancer Risk Assessment and Evaluation for Hereditary Cancer Risk. Best Pract. Res. Clin. Obstet. Gynaecol..

[70] Tyrer J., Duffy S.W., Cuzick J. (2004). A Breast Cancer Prediction Model Incorporating Familial and Personal Risk Factors. Stat. Med..

[71] Lee A., Mavaddat N., Wilcox A.N., Cunningham A.P., Carver T., Hartley S., Villiers C.B.d., Izquierdo A., Simard J., Schmidt M.K. (2019). BOADICEA: A Comprehensive Breast Cancer Risk Prediction Model Incorporating Genetic and Nongenetic Risk Factors. Genet. Med..

[72] Carver T., Hartley S., Lee A., Cunningham A.P., Archer S., Babb de Villiers C., Roberts J., Ruston R., Walter F.M., Tischkowitz M. (2021). CanRisk Tool—A Web Interface for the Prediction of Breast and Ovarian Cancer Risk and the Likelihood of Carrying Genetic Pathogenic Variants. Cancer Epidemiol. Biomark. Prev..

[73] Lindor N.M., Johnson K.J., Harvey H., Shane Pankratz V., Domchek S.M., Hunt K., Wilson M., Cathie Smith M., Couch F. (2010). Predicting BRCA1 and BRCA2 Gene Mutation Carriers: Comparison of PENN II Model to Previous Study. Fam. Cancer.

[74] Panchal S.M., Ennis M., Canon S., Bordeleau L.J. (2008). Selecting a BRCA Risk Assessment Model for Use in a Familial Cancer Clinic. BMC Med. Genet..

[75] Torkamani A., Wineinger N.E., Topol E.J. (2018). The Personal and Clinical Utility of Polygenic Risk Scores. Nat. Rev. Genet..

[76] Osler T.S., Schoeman M., Pretorius W.J.S., Mathew C.G., Edge J., Urban M.F. (2025). Application of Genetic Testing Criteria for Hereditary Breast Cancer in South Africa. Breast Cancer Res. Treat..

[77] Peterson J.M., Pepin A., Thomas R., Biagi T., Stark E., Sparks A.D., Johnson K., Kaltman R. (2020). Racial Disparities in Breast Cancer Hereditary Risk Assessment Referrals. J. Genet. Couns..

[78] Schwartz C., Chukwudozie I.B., Tejeda S., Vijayasiri G., Abraham I., Remo M., Shah H.A., Rojas M., Carillo A., Moreno L. (2021). Association of Population Screening for Breast Cancer Risk With Use of Mammography Among Women in Medically Underserved Racial and Ethnic Minority Groups. JAMA Netw. Open.

