# Supplementary material for: The Current State of Breast Cancer Genetics in Populations of African Ancestry
Source: Genes (Basel). 2025 Feb 6;16(2):199. doi: 10.3390/genes16020199 (PMC11855290; doi:10.3390/genes16020199)
Supplement: Supplementary file 1 [file genes-16-00199-s001.zip › genes-3390331-supplementary.pdf]

Supplementary Table S1 – Summary of articles used in our research.

TITLE	JOURNAL	YEAR	FIRST AUTOR	DOI
		**Admixed Populations**		
Admixture in Latin America	Current opinion in genetics & development	2016	ADHIKARI, K.	10.1016/j.gde.2016.09.003
BROCA gene panel testing in African descendants from northeastern Brazil: Genetic susceptibility profile of an admixed population	Journal of Clinical Oncology	2017	FELIX, G. E. S.	10.1200/JCO.2017.35.15_suppl.1572
Genetic ancestry of 1127 Brazilian breast cancer patients and its correlation with molecular subtype and geographic region	Clinical Breast Cancer	2023	DA COSTA VIEIRA, R. A.	10.1016/j.clbc.2023.04.001
Genomic Diversity in Sporadic Breast Cancer in a Latin American Population	Genes	2020	BRIGNONI, L.	10.3390/genes11111272
Identification of Variants (rs11571707, rs144848, and rs11571769) in the *BRCA2* Gene Associated with Hereditary Breast Cancer in Indigenous Populations of the Brazilian Amazon.	Genes	2021	DOBBIN, E. A. F.	10.3390/genes12020142
Mutational spectrum of breast cancer susceptibility genes among women ascertained in a cancer risk clinic in Northeast Brazil	Breast Cancer Research and Treatment	2022	FELIX, G. E. S.	10.1007/s10549-022-06560-0
Origin and dynamics of admixture in Brazilians and its effect on the pattern of deleterious mutations	PNAS	2015	KEHDY, F. S. G.	10.1073/pnas.1504447112
Prevalence of *BRCA1*/*BRCA2* mutations in a Brazilian population sample at-risk for hereditary breast cancer and characterization of its genetic ancestry	Oncotarget	2016	FERNANDES, G. C.	10.18632/oncotarget.12610
Role of Polygenic Risk Score in Cancer Precision Medicine of Non-European Populations: A Systematic Review	Current Oncology	2022	JUNIOR, H. L. R.	10.3390/curroncol29080436
		**African Populations**		
A Review of Cancer Genetics and Genomics Studies in Africa	Frontiers in Oncology	2021	ROTIMI, S. O.	10.3389/fonc.2020.606400
African Ancestry-Associated Gene Expression Profiles in Triple-Negative Breast Cancer Underlie Altered Tumor Biology and Clinical Outcome in Women of African Descent	Cancer Discovery	2022	MARTINI, R.	10.1158/2159-8290.CD-22-0138
Age at Breast Cancer Diagnosis in Populations of African and European Ancestry	The Breast Journal	2014	KADHEL, P.	10.1111/tbj.12228

Cancer in Africa: The Untold Story	Frontiers in Oncology	2021	HAMDI, Y.	10.3389/fonc.2021.650117
Cancer statistics for African American/Black People 2022	CA: A Cancer Journal for Clinicians	2022	GIAQUINTO, A. N.	10.3322/caac.21718
Clinicopathological and Molecular Study of Triple-Negative Breast Cancer in Algerian Patients	Pathology & Oncology Research	2018	GACEB, H.	10.1007/s12253-017-0242-2
Comparative Analysis of Breast Cancer Phenotypes in African American, White American, and West Versus East African patients: Correlation Between African Ancestry and Triple-Negative Breast Cancer	Annals of Surgical Oncology	2016	JIAGGE, E.	10.1245/s10434-016-5420-z
Contribution of BRCA1 and BRCA2 Germline Mutations to Early Algerian Breast Cancer	Disease Markers	2016	HENOUDA, S.	10.1155/2016/7869095
Epidemiology of Breast Cancer in Sub-Saharan Africa	Breast Cancer Updates	2023	ADEOYE, P. A.	10.5772/intechopen.109361
Genetics of breast cancer in African populations: a literature review	Global health, epidemiology and genomics	2018	ABBAD, A.	10.1017/gheg.2018.8
Genome-wide association studies in women of African ancestry identified 3q26.21 as a novel susceptibility locus for oestrogen receptor negative breast cancer	Human Molecular Genetics	2016	HUO, D.	10.1093/hmg/ddw305
Prevalence of Inherited Mutations in Breast Cancer Predisposition Genes among Women in Uganda and Cameroon	Cancer Epidemiology, Biomarkers & Prevention	2020	ADEDOKUN, B.	10.1158/1055-9965.EPI-19-0506
Rising global burden of breast cancer: the case of sub-Saharan Africa (with emphasis on Nigeria) and implications for regional development: a review	World Journal of Surgical Oncology	2018	AZUBUIKE, S. O.	10.1186/s12957-018-1345-2
Triple-Negative Breast Cancer, Stem Cells, and African Ancestry	The American Journal of Pathology	2018	JIAGGE, E.	10.1016/j.ajpath.2017.06.020
BRCA1 and BRCA2 Gene Mutations in Breast Cancer among West African Women	African Journal of Biomedical Research	2018	ZOURÉ, A.A.	https://www.ajol.info/index.php/ajbr/article/view/165958
African ancestry and higher prevalence of triple-negative breast cancer: findings from an international study	Cancer	2010	STARK, A.	10.1002/cncr.25276
An enhancer variant associated with breast cancer susceptibility in Black women regulates *TNFSF10* expression and antitumor immunity in triple-negative breast cancer	Human Molecular Genetics	2023	HAN, Y.J.	10.1093/hmg/ddac168

Breast cancer disparities in outcomes; unmasking biological determinants associated with racial and genetic diversity	Clinical & Experimental Metastasis	2022	MARTINI, R.	10.1007/s10585-021-10087-x
Neighborhood Disadvantage, African Genetic Ancestry, Cancer Subtype, and Mortality Among Breast Cancer Survivors	JAMA Network Open	2023	IYER, H.S.	10.1001/jamanetworkopen.2023.31295
Prevalence and prognosis of molecular phenotypes in breast cancer patients by age: a population-based retrospective cohort study in western Algeria	Pan African Medical Journal	2021	BELHADJ, A.	10.11604/pamj.2021.38.88.21370
Health Disparities and Triple-Negative Breast Cancer in African American Women: A Review	JAMA surgery	2017	NEWMAN, L. A.	10.1001/jamasurg.2017.0005
Analysis of the genomic landscapes of Barbadian and Nigerian women with triple negative breast cancer	Cancer causes & control	2022	HERCULES, S. M.	10.1007/s10552-022-01574-x
Differences in microRNA expression in breast cancer between women of African and European ancestry	Carcinogenesis	2019	GONG, Z.	10.1093/carcin/bgy134
Epidemiology, biology, and treatment of triple-negative breast cancer in women of African ancestry	The Lancet Oncology	2014	BREWSTER, A. M.	10.1016/S1470-2045(14)70364-X
Common variants on chromosomes 2q35 and 16q12 confer susceptibility to estrogen receptor–positive breast cancer	Nature Genetics	2007	STACEY, S.N.	10.1038/ng2064
Genome-wide association analyses of breast cancer in women of African ancestry identify new susceptibility loci and improve risk prediction	Nature Genetics	2024	JIA, G.	10.1038/s41588-024-01736-4
Breast cancer statistics 2024	CA: A Cancer Journal for Clinicians	2024	GIAQUINTO, A. N.	10.3322/caac.21863
Global cancer statistics 2022: GLOBOCAN estimates of incidence and mortality worldwide for 36 cancers in 185 countries	CA: A Cancer Journal for Clinicians	2024	BRAY, F.	10.3322/caac.21834
**Other Populations**				
Association of breast cancer risk with genetic variants showing differential allelic expression: Identification of a novel breast cancer susceptibility locus at 4q21	Oncotarget	2016	HAMDI, Y.	10.18632/oncotarget.12818
Breast cancer	Nature Reviews Disease Primers	2019	HARBECK, N.	10.1038/s41572-019-0111-2
Breast Cancer Disparities: how can we leverage genomics to improve outcomes?	Surgical Oncology Clinics	2018	DAVIS, M. B.	10.1016/j.soc.2017.07.009
Comparison of Treatment Costs for Breast Cancer, by Tumor Stage and Type of Service	American Health & Drug Benefits	2016	BLUMEN, H.	https://www.ncbi.nlm.nih.gov/pmc/articles

				/PMC4822976/
Discovery of structural deletions in breast cancer predisposition genes using whole genome sequencing data from > 2000 women of African-ancestry	Human Genetics	2021	CHEN, Z.	10.1007/s00439-021-02342-8
Epidemiology of Breast Cancer in Europe and Africa	Journal of Cancer Epidemiology	2012	ABDULRAHMAN, G.O.	10.1155/2012/915610
Estimates and Projections of the Global Economic Cost of 29 Cancers in 204 Countries and Territories From 2020 to 2050	JAMA Oncology	2023	CHEN, S.	10.1001/jamaoncol.2022.7826
Inflammatory breast cancer: Clinical progress and the main problems that must be addressed	Breast Cancer Research	2003	GIORDANO, S. H.	10.1186/bcr608
Nuclear location and cell cycle regulation of the BRCA2 protein	Cancer Research	1997	BERTWISTLE, D.	https://pubmed.ncbi.nlm.nih.gov/9407955/
Racial and Ethnic Disparities Among Participants in Precision Oncology Clinical Studies	JAMA Network Open	2021	ALDRIGHETTI, C. M.	10.1001/jamanetworkopen.2021.33205
Review of cancer from perspective of molecular	Journal of Cancer Research and Practice	2017	HASSANPOUR, S. H.	10.1016/j.jcrpr.2017.07.001
Strategies for subtypes—dealing with the diversity of breast cancer: highlights of the St Gallen International Expert Consensus on the Primary Therapy of Early Breast Cancer 2011	Annals of Oncology	2011	GOLDHIRSCH, A.	10.1093/annonc/mdr304
The impact of race and ethnicity in breast cancer—disparities and implications for precision oncology	BMC Medicine	2022	HIRKO, K. A.	10.1186/s12916-022-02260-0
Common variants on chromosome 5p12 confer susceptibility to estrogen receptor–positive breast cancer	Nature Genetics	2008	STACEY, S. N.	10.1038/ng.131
Triple Negative Breast Cancer Treatment Options and Limitations: Future Outlook	Pharmaceutics	2023	OBIDIRO, O.	10.3390/pharmaceutics15071796
Oncogenic pathways in hereditary and sporadic breast cancer	Maturitas	2004	KENEMANS, P.	10.1016/j.maturitas.2004.06.005
Screening of *HELQ* in breast and ovarian cancer families	Familial Cancer	2016	PELTTARI, L. M.	10.1007/s10689-015-9838-4
Breast Cancer—Epidemiology, Classification, Pathogenesis and Treatment (Review of Literature)	Cancers	2022	SMOLARZ, B.	10.3390/cancers14102569
Global Cancer Statistics 2020: GLOBOCAN Estimates of Incidence and Mortality Worldwide for 36 Cancers in 185 Countries	CA: A Cancer Journal for Clinicians	2021	SUNG, H.	10.3322/caac.21660

Distinction between hereditary and sporadic breast cancer on the basis of clinicopathological data	Journal of Clinical Pathology	2006	VAN DER GROEP, P.	10.1136/jcp.2005.032151
Advanced Approaches to Breast Cancer Classification and Diagnosis	Frontiers in Pharmacology	2021	ZUBAIR, M.	10.3389/fphar.2020.632079

Supplementary Table S2. Summary of breast cancer GWAS with African or African Ancestry Populations

TITLE	JOURNAL, YEAR	FIRST AUTOR	COHORT	DOI
Cross-ancestry GWAS meta-analysis identifies six breast cancer loci in African and European ancestry women	Nature Communications, 2021	Adedokun, B.	African (Ghana and Nigeria), African-Ancestry (Barbados and U.S.), and European	10.1038/s41467-021-24327-x
A survey of microRNA single nucleotide polymorphisms identifies novel breast cancer susceptibility loci in a case-control, population-based study of African-American women	Breast cancer research, 2018	Bensen, J.T.	African American	10.1186/s13058-018-0964-4
A genome-wide association study of breast cancer in women of African ancestry	Human Genetics, 2013	Chen, F.	African American and Afro-Caribbean	10.1007/s00439-012-1214-y
SLCO1B1 polymorphisms and plasma estrone conjugates in postmenopausal women with ER+ breast cancer: genome-wide association studies of the estrone pathway	Breast Cancer Research and Treatment, 2017	Dudenkov, T.M.	Asian, African American and European	10.1007/s10549-017-4243-3
Anastrozole Aromatase Inhibitor Plasma Drug Concentration Genome-Wide Association Study: Functional Epistatic Interaction between *SLC38A7* and *ALPPL2*	Clinical Pharmacology and Therapeutics, 2019	Dudenkov, T.M.	European, Asian, and African American	10.1002/cpt.1359
Polygenic Risk Scores for Prediction of Breast Cancer Risk in Women of African Ancestry: a Cross-Ancestry Approach	Human Molecular Genetics, 2022	Gao, G.	African American, Afro-Caribbean (Barbados), Ghana and Nigeria	10.1093/hmg/ddac102
A common variant at the *TERT-CLPTM1L* locus is associated with estrogen receptor-negative breast cancer	Nature Genetics, 2011	Haiman, C.A.	European and African American	10.1038/ng.985
Genome-wide association studies in women of African ancestry identified 3q26.21 as a novel susceptibility locus for oestrogen receptor negative breast cancer	Human Molecular Genetics, 2016	Huo, D.	African American, Afro-Caribbean and Nigeria	10.1093/hmg/ddw305
Genetic Polymorphisms in the Long Noncoding RNA MIR2052HG Offer a Pharmacogenomic Basis for the Response of Breast Cancer	Cancer Research, 2016	Ingle, J.N.	European, African American and Asian	10.1158/0008-5472.CAN-16-1371

Patients to Aromatase Inhibitor Therapy				
Genome-wide association analyses of breast cancer in women of African ancestry identify new susceptibility loci and improve risk prediction	Nature Genetics, 2024	Jia, G.	African American, Afro-Caribbean (Barbados), Ghana, Nigeria, Uganda, and Cameroon	10.1038/s41588-024-01736-4
Cross-ancestry GWAS defines the extended *CYP2D6* locus as the principal genetic determinant of endoxifen plasma concentrations	Clinical Pharmacology and Therapeutics, 2023	Khor, C.C.	Asian, Lebanese ancestry, European	10.1002/cpt.2846
A pilot genome-wide association study of early-onset breast cancer	Breast Cancer Research and Treatment, 2009	Kibriya, M.G.	European ancestry, Hispanic, African American	10.1007/s10549-008-0039-9
Genome-wide enriched pathway analysis of acute post-radiotherapy pain in breast cancer patients: a prospective cohort study	Human Genomics, 2019	Lee, E.	Hispanic, European ancestry, African American	10.1186/s40246-019-0212-8
Genome-Wide Association Studies for Taxane-Induced Peripheral Neuropathy in ECOG-5103 and ECOG-1199	Clinical cancer research, 2015	Schneider, B.P.	African American and European ancestry	10.1158/1078-0432.CCR-15-0586
A meta-analysis of genome-wide association studies of breast cancer identifies two novel susceptibility loci at 6q14 and 20q11	Human Molecular Genetics, 2012	Siddiq, A.	European ancestry and African American	10.1093/hmg/dds381
A genome-wide scan for breast cancer risk haplotypes among African American women	PloS One, 2013	Song, C.	African American	10.1371/journal.pone.0057298
Genome-wide meta-analyses identifies novel taxane-induced peripheral neuropathy-associated loci	Pharmacogenetics and genomics, 2018	Sucheston-Campbell, L.E.	European ancestry and African American	10.1097/FPC.0000000000000318
Germline variants and somatic mutation signatures of breast cancer across populations of African and European ancestry in the US and Nigeria	International Journal of Cancer, 2019	Wang, S.	African American, Asian, and European ancestry	10.1002/ijc.32498
UACA locus is associated with breast cancer chemoresistance and survival	NPJ Breast Cancer, 2022	Zhu, Q.	European, East Asian, Hispanic, African American	10.1038/s41523-022-00401-5

Supplementary Table S3 – Summary of the most relevant SNPs found on Jia G. GWAS [62]

SNP	Subtype	OR (95% CI)	Nearby Gene	Gene Region	Observations
**rs76664032**	TNBC	1.30 (1.20-1.42)	*RP11-19E11.1*	10 kb from 3'-UTR	Common risk allele (81%), located near a lncRNA gene. eQTL analyses suggest an association with lower expression of RP11-19E11.1.
**rs10069690**	TNBC, ER-	1.38 (1.30-1.47)	*TERT*	Intronic	Also associated with TNBC risk in women of European ancestry but with weaker associations. Risk allele frequency is higher in African populations.
**rs12974508**	TNBC, ER-	1.38 (1.29-1.47)	*ABHD8*	Intergenic	
**rs10853615**	ER+	1.15 (1.10-1.21)	*TTC39C*	Intronic	No previous studies have found associations between *TTC39C* and breast cancer.
**rs61751053**	Overall BC	1.48 (1.30-1.70)	*ARHGEF38*	Missense	Low-frequency variant, risk allele rare in Asian and European populations.
